# Development of a Discriminative Dissolution Method, Using In-Silico Tool for Hydrochlorothiazide and Valsartan Tablets

**DOI:** 10.3390/pharmaceutics15061735

**Published:** 2023-06-14

**Authors:** Rosmery Merma Leon, Michele Georges Issa, Marcelo Dutra Duque, Josiane Souza Pereira Daniel, Humberto Gomes Ferraz

**Affiliations:** 1 Department of Pharmacy, Faculty of Pharmaceutical Sciences, Universidade de São Paulo—USP, Av. Prof. Lineu Prestes, 580, São Paulo 05508-080, SP, Brazil; rosmerymerma@gmail.com (R.M.L.); sferraz@usp.br (H.G.F.); 2 Department of Pharmaceutical Sciences, Institute of Environmental, Chemical and Pharmaceutical Sciences, Universidade Federal de São Paulo—UNIFESP, Rua São Nicolau, 210 Centro, Diadema 09913-030, SP, Brazil; marcelo.duque@unifesp.br

**Keywords:** dissolution, in silico, simulations, DDDPlus, hydrochlorothiazide, valsartan, factorial design

## Abstract

Hydrochlorothiazide (HTZ) and Valsartan (VAL) are poorly soluble drugs in BCS classes IV and II. This study aimed to develop a method to assess the dissolution profile of tablets containing HTZ (12.5 mg) and VAL (160 mg) as a fixed-dose combination, using in silico tools to evaluate products marketed in Brazil and Peru. Firstly, in vitro dissolution tests were performed using a fractional factorial design 3^3−1^. Then, DDDPlus™ was used to carry out experimental design assays of a complete factorial design 3^3^. Data from the first stage were used to obtain calibration constants for in silico simulations. The factors used in both designs were formulation, sinker use, and rotation speed. Finally, effects and factor interaction assessment was evaluated based on a statistical analysis of the dissolution efficiency (DE) obtained from simulations. Thus, the established final conditions of the dissolution method were 900 mL of phosphate buffer pH 6.8, 75 rpm of rotation speed, and sinker use to prevent formulation floating. The reference product stood out because of its higher DE than other formulations. It was concluded that the proposed method, in addition to ensuring total HTZ and VAL release from formulations, has adequate discriminative power.

## 1. Introduction

Drug dissolution profiles for solid oral dosage forms must be assessed to demonstrate their performance. For this, in addition to the sink condition, the method must be sufficiently discriminative to exclude formulations that do not meet the criteria for appropriate in vivo performance [1,2]. The challenge in developing a dissolution method is more significant when involving BCS classes II and IV drugs in fixed-dose combination formulations, which is essential to ensure that the dissolution of one drug does not interfere with the solubilization of the other compound in the dissolution medium [3].

A fundamental question for dissolution method development is the knowledge of the solubility of the drug in the physiological pH range. Among the methods that can be used to determine solubility, potentiometric titration proves to be quite interesting when compared with the traditional shake-flask. This is due to the reduced sample required (2 to 10 mg) and the time to obtain the result [4].

Using this technique, drug solubility is determined by the concentration of neutral molecules in the solution, which can be calculated using the measured balanced pH and drug pKa. The solubility logarithm versus pH curve is provided by the equipment software just after titration time [4].

Additionally, the combination of statistical experimental design and modeling & simulation tools has been demonstrated as an exciting approach to formulation development [5,6] that can also be used for dissolution method assessment.

Computer simulations are very useful tools that, in addition to providing reliable and fast results, help reduce the number of in vitro assays. For example, computer software DDDPlus™ (Simulations Plus, Lancaster, CA, USA) can simulate dissolution assays of drug-containing formulations based on data such as molecule physicochemical properties, excipients used, and dissolution apparatus [5,7,8].

Hydrochlorothiazide (HTZ) is a weak acid drug with pKa 7.90, and its structure contains chloride in the ortho position relative to Sulfonamide, which makes the drug more liposoluble [9]. Valsartan (VAL) is a weak acid composed of a tetrazole and carboxyl group with pKa 4.73 and 3.90, respectively. The carboxylic acid group is responsible for the low solubility of this drug at low pH, which is why VAL is defined as a pH-dependent solubility drug [10]. Both are poorly soluble drugs in aqueous media and belong to BCS Classes IV and II. These drugs are marketed in Brazil and Peru in a fixed-dose combination of VAL 160 mg + HTZ 12.5 mg.

The objective of this study was to develop a discriminative dissolution methodology to assess the dissolution profile of fixed-dose combination formulations containing VAL (160 mg) plus HTZ (12.5 mg) using in silico tool DDDPlus™ through experimental factorial design and subsequently assess dissolution profiles from products marketed in Brazil and Peru.

## 2. Materials and Methods

### 2.1. Materials

Valsartan (101.2% purity on a dry basis) and hydrochlorothiazide (100.1% purity on a dry basis) were kindly provided by Aché Laboratórios SA (Guarulhos, São Paulo, Brazil).

Purified water was obtained using the Milli-Q filtration system (Merck Millipore, Darmstadt, Germany), and the reagents 0.15 M potassium chloride (KCl), 0.5 M potassium hydroxide (KOH) and methanol (LabSynth, Diadema, Brazil) were used in the solubility assay.

Purified water and polysorbate 80 (Sigma Aldrich, São Paulo, Brazil) were used in the particle size analysis.

Formulations for the dissolution method development were prepared using the following excipients: PH 200 microcrystalline cellulose—Avicel PH 200 LM NF (FMC Corporation, Philadelphia, PA, USA), PH 302 microcrystalline cellulose—Avicel PH 302 NF (FMC Corporation, Philadelphia, PA, USA), croscarmellose sodium (Blanver Farmoquímica LTDA, Taboão da Serra, Brazil), colloidal silicon dioxide—Aerosil 200 (Henrifarma Produtos Químicos e Farmacêuticos Ltd., São Paulo, Brazil), lactose monohydrate—Tablettose^®^ 70 (Meggle Pharma, Wasserburg, Germany), magnesium stearate (Meggle Pharma, Wasserburg, Germany).

The reagents used in tablet content analysis were purified water by Milli-Q system (Merck Millipore, Darmstadt, Germany), ammonium formate (97%) (Sigma Aldrich, São Paulo, Brazil), acetonitrile and high-performance liquid chromatography (HPLC)-grade methanol (Merck, São Paulo, Brazil).

Dissolution media were prepared using purified water obtained by reverse osmosis, monobasic potassium phosphate (LabSynth, Diadema, Brazil) and sodium hydroxide (LabSynth, Diadema, SP, Brazil).

Formulations containing VAL (160 mg) + HTZ (12.5 mg) in tablet and capsule presentations were purchased at drugstores in Brazil and Peru. The drug products were designated as Reference drug products, B1 and B2 (coated tablets marketed in Brazil) and P2 (coated tablets), and C1 and P1 (capsules) from Peru.

### 2.2. Solubility Assay

Solubility assay was performed using an acid-base titration (pH range 2–12) potentiometric method on Sirius T3 Automatic Titration System (Sirius Analytical Instruments Ltd., East Sussex, UK) with Ag/AgCl pH electrode using a 10 mg sample carefully weighed on AUW220D analytical balance (Shimadzu, Japan). First, the ionic strength was adjusted with 1.5 mL solution at 0.15 M KCl at 37.0 ± 0.5 °C with constant rotation and under a nitrogen-based atmosphere. Then, small amounts of acid and base were added alternately to repeatedly dissolve and precipitate the material until the balance was achieved between the drug’s molecular and ionized states.

VAL and HTZ drugs pKa values were also determined using this triplicate technique with a 1-mg sample, and methanol was used as a co-solvent (20%, 30%, and 40% *v*/*v*). Data obtained from water-methanol mixtures were extrapolated to aqueous conditions by refinement as per the Yasuda-Shedlovsky equation [11,12] using Sirius T3 Version 1.1.2.0 software.

HTZ potentiometric titration was conducted by the Chasing equilibrium method—Cheqsol [4] and the curve fitting method for VAL. The results obtained for HTZ and VAL were calculated and refined using the Sirius T3 Refine Version 1.1.2.0 software.

### 2.3. Drug Particle Size

During the wet method, particle size distribution was carried out using Granulometer Cilas 1900 laser diffractometer (Cilas, Orleans, France). HTZ was dispersed in purified water, and the suspension obtained was submitted to agitation in the equipment without sonication. The measurement of the particle size was done every 30 s. The dispersing medium was purified for VAL with three drops of polysorbate 80. The mixture was inserted in the dispersion unit of the equipment and submitted to agitation and sonication with measurements of the particle size every 30 s. In both cases, the amount of dispersion analyzed was sufficient to maintain an adequate obscuration range (10–30%).

### 2.4. Formulations for Dissolution Method Development

The composition of three different formulations obtained by direct compression is present in Table 1.

To prepare each formulation, the components were mixed in a polyethene bag for 5 min, and then magnesium stearate was added and mixed for an additional 1 min. The mixture was subjected to direct compression in a hydraulic press (América Lab., São Paulo, Brazil), with a pressure of 1000 psi, punch 12 mm in diameter for 20 s.

#### 2.4.1. Tablet Disintegration

A disintegration assay was performed as described in the Brazilian Pharmacopeia 6th Edition (2019) [13], using 301-disintegrator equipment (Ethik Technology, São Paulo, Brazil). Six units of each formulation were tested in phosphate buffer pH 6.8 (750 mL) at 37 ± 0.5 °C with discs to reduce variability in results.

#### 2.4.2. Tablet Content

The content of VAL and HTZ drugs was determined using quantification by HPLC, using a Sunfire BEH C8 1.7 µm, 2.1, 100 mm column, in 0.01 M ammonium formate gradient pH 4.77 (Table S1) and acetonitrile, with 0.9 mL/min mobile phase flow at 40 °C and 230 nm. UHPLC Waters Acquity equipment (Waters Corporation, Milford, CT, USA), equipped with a photodiode array detector (DAD), was used, and data were collected using EMPOWER version 3 software (Waters Corporation, Milford, CT, USA).

Twenty tablets were weighed and crushed to obtain a fine powder, and then amounts equivalent to 12.5 mg HTZ and 160 mg VAL were weighed and added to a volumetric flask. The first dilution was conducted in methanol, and subsequent dilutions using phosphate buffer pH 6.8 were conducted until final concentrations of 6.5 µg/mL for HTZ and 80 µg/mL for VAL were obtained. Samples were filtered using 0.45-µm polyvinylidene difluoride (PVDF) syringe filters. Drug content was expressed in mg and as a theoretical content percentage. The method was internally validated per Q2R2 ICH Guideline (Supplementary Material).

### 2.5. Dissolution Method Development

The dissolution method was developed using an in vitro and silico experimental design.

In vitro dissolution assays were performed in triplicate, using apparatus 2 (paddle) according to the United States Pharmacopoeia 43-NF38 Valsartan and Hydrochlorothiazide Tablets monograph [14], coupled to Agilent Technologies 708 DS dissolution equipment (Agilent Technologies, Santa Clara, CA, USA), in vessels containing 900 mL of 0.05 M phosphate buffer pH 6.8 as dissolution media at 37 ± 0.5 °C. Sinkers and rotation speed were used according to the statistical experimental design presented in Section 2.5.1. The dissolution tests were conducted during 60 min, with 5-mL aliquots being collected at 5, 10, 15, 20, 30, 45 and 60 min, and filtered using PVDF 0.45-µm syringe filter.

The quantification of the dissolved drugs was performed as described in Section 2.4.2, and the dissolution profiles were constructed for each drug (HTZ and VAL). From these profiles, dissolution efficiencies (DE) were calculated using the Microsoft Excel add-in DDSolver [15].

#### 2.5.1. In Vitro Experimental Design for Dissolution Method Development

For in vitro development, a 3^3−1^ fractional factorial-type experimental design was carried out using Statistica^®^ version 13.0 software (Tibco Software Inc., Palo Alto, CA, USA). The three independent factors were: rotation speed, formulations, and sinkers at three levels each, as shown in Table 2. The experimental matrix design is presented in Supplementary Material as Table S2.

#### 2.5.2. In Silico Experimental Design for Dissolution Method Development

The software used for in silico assays was DDDPlus™ version 5.0 (Simulations Plus, Lancaster, CA, USA). A full 3^3^ factorial design was carried out using factors and levels described in Table 2, resulting in the 27 experiments shown in Table S3.

Data regarding the physicochemical characteristics of active ingredients needed to construct the dissolution model are described in Table 3. Solubility and true density were obtained experimentally, while the others were calculated from HTZ and VAL chemical structure in DDDPlus™ by ADMET Predictor^®^.

The experimental dissolution profiles, obtained as described in Section 2.5.1, were used as inputs in DDDPlus™ to obtain the calibration constants of the mathematical equations of the Nernst-Brunner (VAL) and Mass Transfer (HTZ) dissolution models available in the software. After this step, the other dissolution experiments (Table S3) were simulated using the fitted dissolution models.

#### 2.5.3. Statistical Analysis

Dissolution efficiencies were considered as responses, and ANOVA and post-hoc Tukey’s test were performed using Statistica^®^ version 13.0 (Tibco Software Inc., Palo Alto, CA, USA) and Minitab 18 (Minitab Inc., State College, PA, USA), respectively.

Additionally, cluster and principal component analysis were performed using DE, mean dissolution time (MDT) and dissolved percentages between 5 and 60 min as original variables. DE and MDT values were obtained from the Excel add-in DD Solver [15]. All data were previously standardized, and the main components used to build the two-dimensional graphs were those with the highest eigenvalues. For cluster analysis, the Euclidean distance was used as a classification algorithm.

### 2.6. Dissolution Profile Evaluation of Marketed Samples

Twelve units of each formulation described in Table 1 were tested. The dissolution assay conditions were: 900 mL of 0.05 M phosphate buffer pH 6.8 as dissolution medium; 75 rpm of rotation speed and sinker in Agilent 708 DS dissolution equipment coupled with USP apparatus II (paddle). During the assay, 5-mL aliquots were taken at 5, 10, 15, 20, 30, 45 and 60 min without dissolution media replacement. Samples were filtered using PVDF 0.45-µm syringe filter. Formulations were also assessed by the dissolution method of the United States Pharmacopoeia [14] corresponding monograph, which uses 1000 mL of 0.05 M phosphate buffer pH 6.8 and 50 rpm of rotation speed to compare the discriminative power and effectiveness of the proposed method. The amount dissolved of HTZ and VAL was quantified according to Section 2.4.2.

## 3. Results and Discussion

### 3.1. Solubility Assay

**Figure 1:**
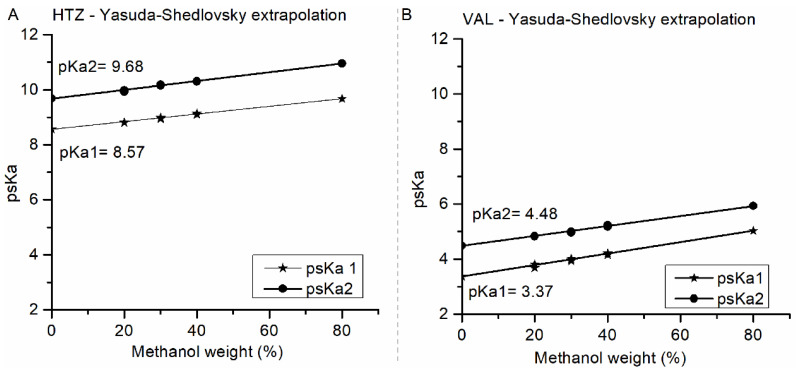
Yasuda-Shedlovsky extrapolation via Sirius T3 version 1.1.2.0 software for (**A**) HTZ and (**B**) VAL: psKa 1 (filled star); psKa 2 (filled circle).

The pKa determination assay was performed with different concentrations of methanol as a co-solvent and led to % methanol versus psKa (dissociation constant with co-solvent) plots, shown in Figure 1. Using Yasuda-Shedlovsky extrapolation, through Sirius T3 Version 1.1.2.0 software, it was possible to calculate drug pKa values for % methanol equal to zero [16].

For HTZ, linear determination coefficients were R^2^ = 0.9872 for psKa1 curve, and R^2^ = 0.9604 for psKa2 curve, while extrapolated pKa values were pKa1 = 8.57 ± 0.02 and pKa2 = 9.68 ± 0.04. For VAL, linear determination coefficients were R^2^ = 0.9303 and R^2^ = 0.9468 for psKa1 and psKa2 curves, respectively, and extrapolated pKa values were pKa1 = 3.37 ± 0.06 and pKa2 = 4.48 ± 0.03 [11]. According to calculated pKa values, HTZ and VAL are weak acidic compounds. In this case, both were dissolved (in alkaline media) and titrated (with acid) until neutral species appeared as a precipitate.

For HTZ, shortly before precipitation, the aqueous solution reached supersaturated conditions relative to neutral species. At this point, using the CheqSol method, the solution was back-titrated with the base until the compound began to dissolve again and reached a sub-saturation state [4]. Based on principles of mass and charge balance, and the constant switch between saturated and unsaturated state, it was possible to find the value of 0.77 mg/mL at pH 5.6 for HTZ, which is close to the value of 0.79 mg/mL described in the literature using similar method [12].

**Figure 2:**
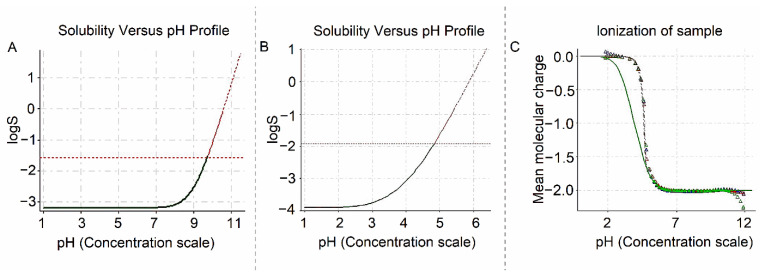
HTZ solubility pH profile (**A**), VAL solubility pH profile (**B**) and respective Bjerrum curve (**C**). Source: Sirius T3 Version 1.1.2.0 software. The dotted line parallel to the *x*-axis in (**A**,**B**) indicates the value of logS to which drug solubility can be measured in acid-base equilibrium.

According to Figure 2A and Table 4, it is possible to verify that there is no significant change in the solubility of HTZ in the physiological range of pH (1.0 to 6.8).

VAL solubility profile (Figure 2B and Table 4) show low solubility under unbuffered or acidic conditions, increasing until complete solubilization above pH 4.5 [10]. VAL titration was conducted using a curve fitting model, in which pH is adjusted in one direction only, and a theoretical Bjerrum curve represents sample precipitation relative to pH (Figure 2B,C). In this case, solubility is manually adjusted to selected data points. Solubility values were measured up to pH 4.84 because when above this value, solubility is influenced by the salt formation in titration media and can be calculated by extrapolation [17].

### 3.2. Drug Particle Size and Tablet Characterization

According to drug solubility, particle size can directly impact the dissolution of solid pharmaceutical dosage forms [18]. Thus, using the experimental value in the software can bring a more mechanistic dissolution model. The average sizes obtained through particle distribution (Figure S1) were 93.51 ± 0.07 µm for HTZ; and 29.25 ± 0.83 µm for VAL and were used as input data in DDDPlus™.

The results of drug content in the formulations were 108.47 ± 1.25%, 108.38 ± 0.73% and 107.30 ± 0.42% for HTZ, and 94.26 ± 0.23%, 98.49 ± 0.39% and 93.58 ± 0.42% for VAL, in the formulations F1, F2 and F3, respectively, which demonstrates that all three formulations comply with the United States Pharmacopeia USP 43-NF38 for HTZ and VAL content specifications (90–110%). In addition, disintegration time was less than 1 min for F3, less than 3 min for F2 and less than 5 min for F1; this is related to differences in types and amounts of excipients used in the formulations. These results are consistent with their composition since F1 does not have a disintegrant; therefore, it takes longer to disintegrate, while F2 and F3, which have 1.0 and 2.0% of disintegrant, respectively, show shorter disintegration time.

### 3.3. Dissolution Method Development

Dissolution conditions defined in the experimental design matrix were performed for HTZ and VAL, respectively (Table 5 and Table 6). According to the United States Pharmacopoeia USP 43 NF 38, fixed-dose combination tablet formulations containing HTZ and VAL must release less than 80% (Q, dissolution percentage) in 30 min of dissolution test [11].

In the results presented for HTZ (Table 5), F1 and F3 were evaluated in the presence of a Japanese basket or sinker (E2, E8, E6, E9) and released more than 85% of the drug within 30 min. For VAL, only in assay E2 (Table 6), which also used a sinker at 50 rpm, drug release was higher than 85% of the drug within 30 min of dissolution.

The use of a sinker is essential in the cases of tablet floating, which can be attributed to its surface degree of hydrophobicity since 50.28% of the tablet corresponds to the low-density drug, and this affects the release mechanism of the drug from the formulation into the dissolution medium [19].

**Figure 3:**
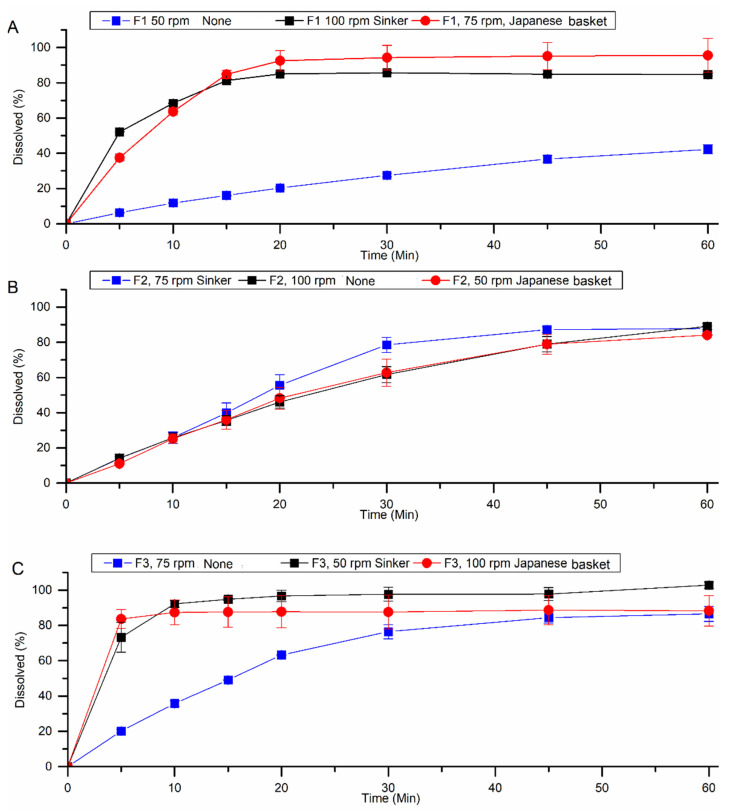
Dissolution profiles of the formulations F1 (**A**), F2 (**B**), and F3 (**C**) were obtained under the test conditions defined in the fractional factorial design for HTZ.

**Figure 4:**
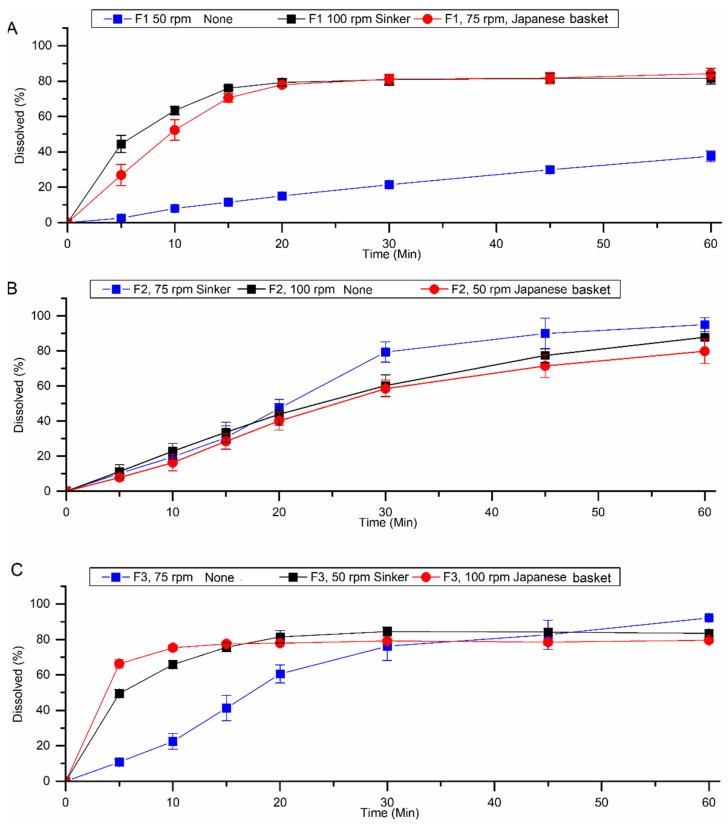
Dissolution profiles of the formulations F1 (**A**), F2 (**B**), and F3 (**C**) were obtained under the test conditions defined in the fractional factorial design for VAL.

As shown in Figure 3 and Figure 4, for formulations F1 and F3, HTZ and VAL release was greatly influenced by the presence of the Japanese basket or sinker. Without it, the formulation released less than 50% of HTZ and VAL due to its floating, which interferes with tablet hydration since the hydrodynamics in the surface of the dissolution vessel is compromised. In assays using formulation F2, the dissolution profile is similar in all three conditions tested. In this case, the diluent used in the formulation was a microcrystalline cellulose PH 302 (Table 1), which presents a higher density and could prevent the tablet from floating. However, it provided a lower release for drugs than F1 and F3.

In the first statistical assessment, observed and predicted residues (Figure S2) from in vitro DE were evaluated for HTZ and VAL; it was observed that DE residues distribution for both drugs, in addition to R^2^ values obtained from the analysis of variance (ANOVA), R^2^ = 0.9793 and R^2^ = 0.9663 for HTZ and VAL DE, respectively, are indicators that the model is appropriate. In addition, the effects of factors formulation, rotation speed, and the presence or absence of sinker for HTZ and VAL were tested using a *p*-value, which must be less than 0.05 if the factor has a significant influence with a 95% confidence level.

**Figure 5:**
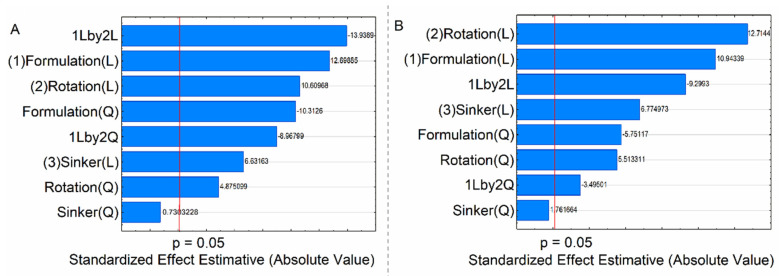
Pareto chart the influence of the factors and their interactions: (**A**) HTZ and (**B**) VAL. Those crossing the vertical (red) line are significant effects.

The Pareto chart (Figure 5) showed that the linear interaction between formulation and rotation speed had the greatest effect on HTZ DE; on the other hand, for VAL, the linear effect of the rotation speed had the greatest significance on DE.

**Figure 6:**
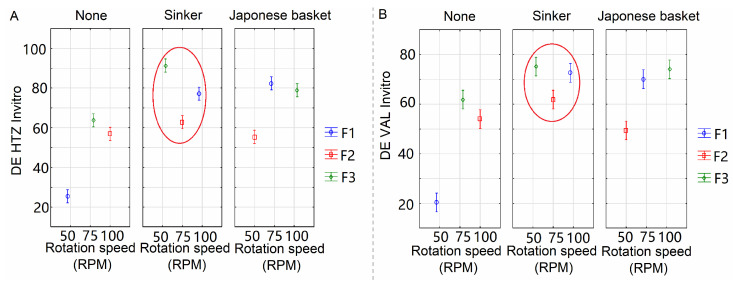
In vitro, development means plotting for interaction between factor effects (rotation speed, sinker use, and formulations) with a 95% confidence level (**A**) HTZ and (**B**) VAL. The red circles represent the approximation of DE values using the sinker.

**Figure 7:**
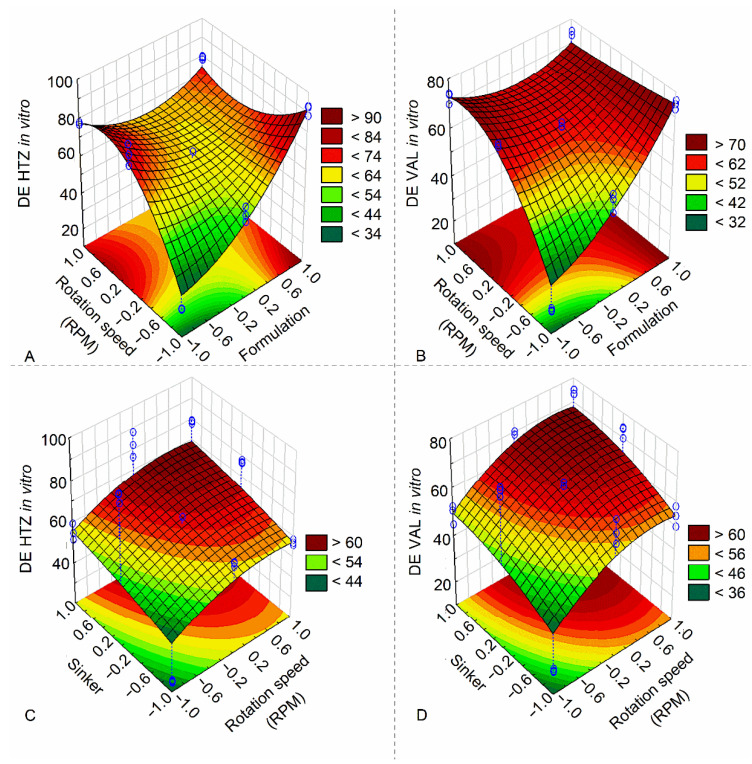
Surface plots of DE effects for HTZ and VAL. (**A**) rotation speed versus formulation for HTZ, (**B**) rotation speed versus formulation for VAL, (**C**) sinker versus rotation speed for HTZ and (**D**) sinker versus rotation speed for VAL.

In Figure 6, it was confirmed that the presence of a sinker and Japanese basket has a positive effect on HTZ and VAL DE. Across assessed formulations, F3 had the highest DE values, which supports its composition (2% croscarmellose; it also contains lactose, which is soluble in water). It is also possible to observe that HTZ and VAL DE values increase proportionally with increased rotation speed from 50 to 75 rpm, and the sinker has a better effect than the Japanese basket; the same effects can be observed in surface plots in Figure 7.

In the in-silico development stage, using DDDPlus™ version 5.0 software, simulated dissolution profiles were obtained for HTZ and VAL, from which DEs were calculated for each profile, in addition to coefficients of determination (R^2^, that shows whether the fit between observed and simulated values is appropriate (Table 7).

According to coefficient of determination (R^2^) values (Table 7), formulation F2 had the best fit between observed and simulated percent dissolution values. This could be explained by observations in the statistical assessment of in vitro assays, where F2 DE was not influenced by the presence of a sinker.

Some simulations with F1 showed a lower coefficient of determination (R^2^ < 0.9); this could be explained by coning during the in vitro dissolution test. However, at low rotation speeds, coning formation with the presence of a sinker or Japanese basket can further alter dissolution hydrodynamics, and this is something that the software cannot yet predict.

A study with another low-solubility drug showed that working with correct data allows simulations to reflect what happens in vitro [20]. Therefore, despite the ADMET Predictor^®^ module available in DDDPlus™ being able to provide results calculated from the drug’s chemical structure, it is often required to perform complementary assays to use as input data in the software and optimize simulations.

**Figure 8:**
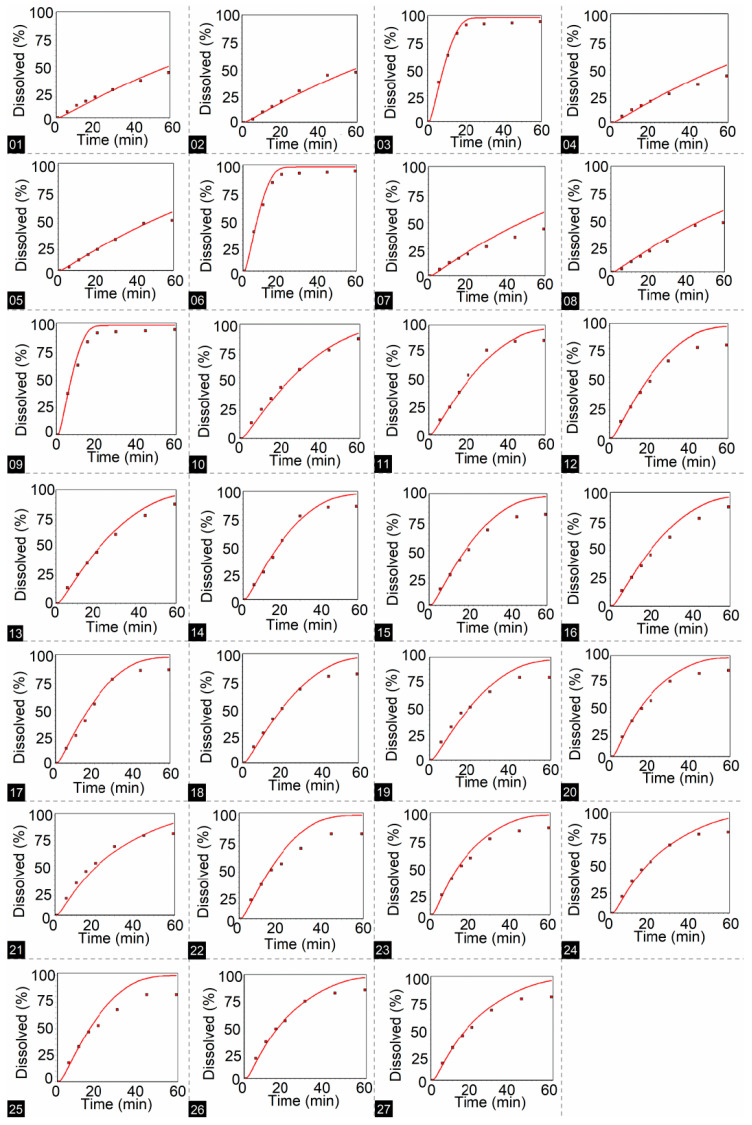
Dissolution profiles for HTZ were obtained using DDDPlus™ according to the full 3^3^ factorial design. The number at the bottom of each figure corresponds to the dissolution test condition (Run) presented in Table 7.

**Figure 9:**
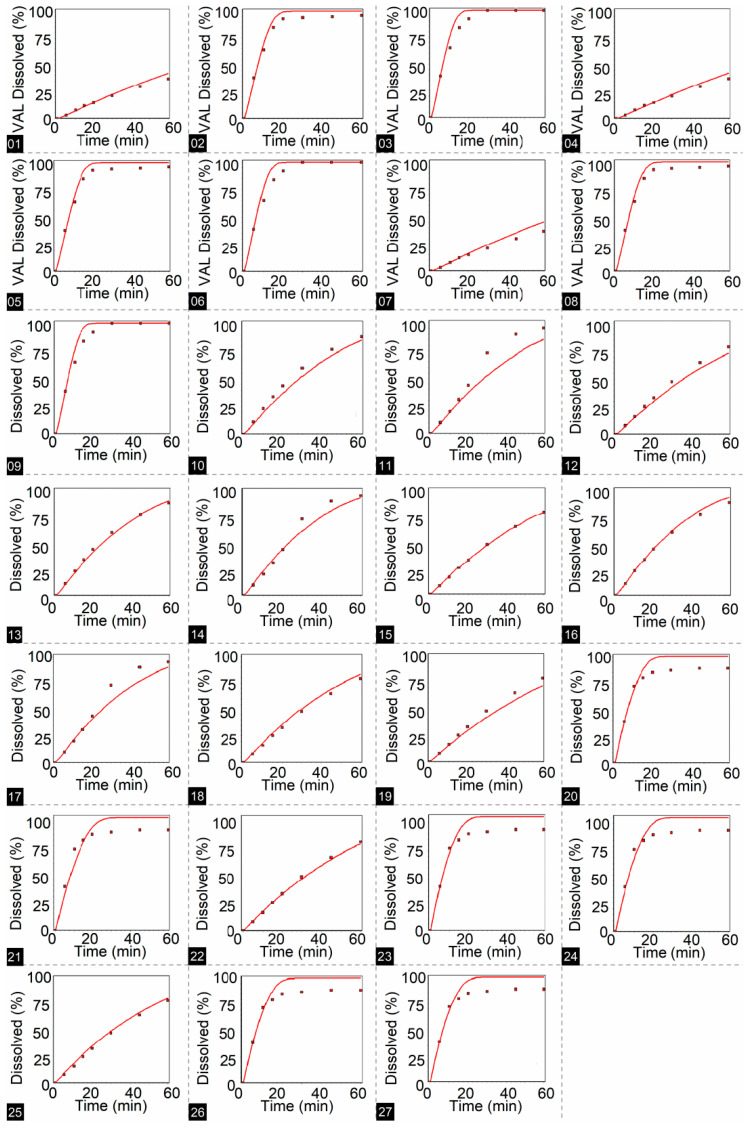
Dissolution profiles for VAL were obtained using DDDPlus™ according to the full 3^3^ factorial design. The number at the bottom of each figure corresponds to the dissolution test condition (Run) presented in Table 7.

Figure 8 and Figure 9 show dissolution profiles across conditions defined in the full factorial design, red dots are the observed percent dissolution, and continuous red lines are simulated points from 0 to 60 min.

Different dissolution results can be obtained according to the size, shape, and density of the entity to be dissolved and its position and distribution in the dissolution vessel. Another factor that can cause differences in dissolution results is a phenomenon called coning that happens more frequently when using apparatus 2. This is a problem often found in dissolution methods development, and if not correctly treated, it can generate false in vitro results. Coning formation can be solved by increasing rotation speed to 75 or 100 rpm or replacing the traditional vessel with a “peak vessel” [8,21,22,23,24].

R^2^ values in HTZ and VAL DE analysis of variance (ANOVA) were R^2^ = 0.9937 and R^2^ = 0.9903, and the distribution of observed versus predicted residues (Figure S3) indicates that the statistical assessment model from in silico development-obtained data is adequate.

The Pareto chart (Figure S4) shows all interactions (linear and quadratic) between factors. Rotation speed influences isolated factors, but the effect in its interaction with the formulation or sinker use is insignificant, which makes a difference in what was observed in DE assessment during experimental dissolution method development, where rotation speed had greater effect on VAL DE. This fact could have been due to the small number of data provided by fractional factorial design, making it impossible to measure all interactions and their effects. Hence, a complete factorial design is often required to understand some processes better. In this case, as this dissolution method involves two drugs, in silico simulation using DDDPlus™ was very useful.

Figure S5 shows the assessment of HTZ and VAL DE means. It can be seen that at 50 rpm, it is impossible to discriminate between formulations, mainly for VAL, although for HTZ, this does not represent a problem for the method. Therefore, the most suitable rotation speed would be 75 rpm, which offers a discriminative power for HTZ and VAL. This was confirmed by the surface plots obtained (Figure S6).

The means plot (Figure S5) reveals that the use of a sinker in its interaction with rotation speeds (50, 75 and 100 rpm) showed the greatest differentiation between formulations F1, F2 and F3, for both HTZ and VAL (Figure S6).

In surface plots (Figure S6), it was observed that HTZ and VAL DE behaved similarly, and DE for all three assessed formulations did not vary significantly with the increased rotation speed.

**Figure 10:**
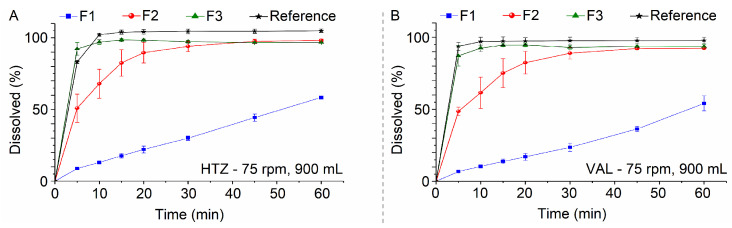
Dissolution profiles of the formulations F1, F2, F3 and the reference drug product, for HTZ (**A**) and VAL (**B**), evaluated using the defined dissolution method: 900 mL of phosphate buffer pH 6.8, apparatus 2 at 75 rpm and sinker.

After the statistical assessment, the conditions defined for the dissolution method were: 900 mL of phosphate buffer pH 6.8 as dissolution media, 75 rpm of rotation speed and sinker use to avoid formulation floating. Once these conditions were defined, the dissolution profiles of all three formulations were assessed to show the discriminative power of the method. Finally, the dissolution profiles were constructed with percent dissolution (Figure 10A,B).

ANOVA was performed using Minitab version 18.1 software (Minitab Inc., State College, PA, USA) for DEs obtained for HTZ and VAL across all three formulations. In both cases, *p*-value < 0.05 was obtained, with R^2^ = 0.9998 and adjusted R^2^ = 0.9997 for HTZ and R^2^ = 0.9963 and adjusted R^2^ = 0.9951 for VAL. This result indicates differences between formulations, so a Tukey’s test was performed (Figure S7). For HTZ and VAL, DEs were grouped into three different groups, thus confirming that the selected method can discriminate between all three formulations.

It was shown that according to changes in formulation concerning diluent and disintegrant levels, there are differences in HTZ and VAL dissolution profiles. Furthermore, changes in disintegrant amounts and their combination with the type of diluent used led to differences in dissolution profiles [25].

Once the discriminative power of the dissolution method is established, it can be used in the early stages of product development to select the best formulation, assess excipients with different quality attributes, and assess small formulation changes [26]. In addition, a well-developed dissolution method is extremely useful in assessing in vitro drug release, indicating which formulations would perform best in subsequent bioequivalence studies [27,28].

In silico modeling and simulation methods can complement formulation development, increasing the probability of formulation success and bioequivalence and bioavailability studies [29]. Therefore, assessing the relationship between solubility, particle size, dissolution, and formulation characteristics is essential to reach the rational development of a discriminative dissolution method that can be practically evaluated in silico modeling using DDDPlus™ [5].

### 3.4. Dissolution Profile of Market Samples with the Developed Method

Formulations content (Table 8) is within acceptable limits established in the United States Pharmacopoeia (90–110%) for both drugs established on the label. Values are expressed in mg/tablet or mg/capsule and percentage relative to dose.

**Figure 11:**
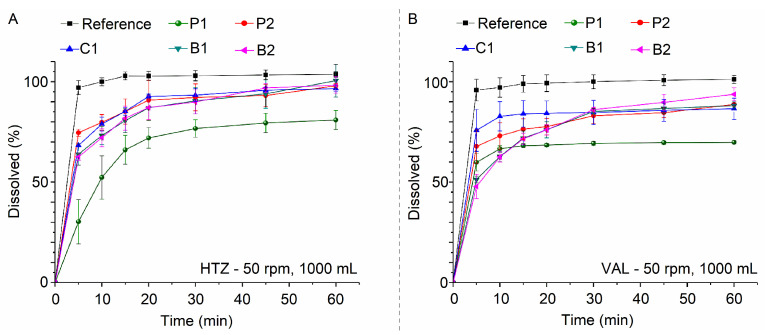
Dissolution profiles of commercial formulations as assessed using the method described in the United States Pharmacopoeia. HTZ (**A**) and VAL (**B**).

Figure 11 shows that the pharmacopeial method led to the complete release of HTZ and VAL in the first 5 min for the reference formulation only. Formulations P2, C1, B1, and B2 released more than 85% HTZ in 30 min, while P1 released 76.66%. Results show that reference products B1 and B2 released more than 85% VAL for VAL release, but P1, P2 and C1 cannot reach the specification. For HTZ and VAL dissolution from fixed-dose combination formulations, at least 80% Q in 30 min is necessary. According to this criterion, P1, P2 and C1 would be rejected products for not satisfying quality control conditions for drug release.

**Figure 12:**
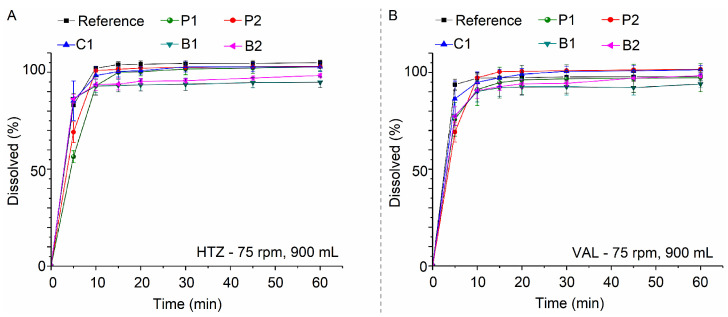
Dissolution profiles of commercial formulations as assessed using the proposed method. HTZ (**A**) and VAL (**B**).

**Figure 13:**
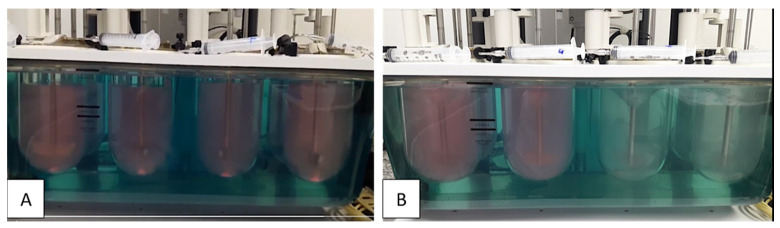
(**A**) Coning formation at the bottom of dissolution vessels at 50 rpm rotation speed, (**B**) coning absent in dissolution vessels at 75 rpm.

With the proposed method, HTZ and VAL mean dissolved percentage at 30 min was 100.16 ± 4.34% and 97.11 ± 3.30%, respectively (Figure 12). However, coning formation is observed in assays performed with the pharmacopeial method (Figure 13A). The method proposed employing 75 rpm solves the coning problem, thus improving the hydrodynamics for HTZ and VAL (Figure 13B), favoring the complete dissolution of HTZ and VAL from formulations.

Demonstrating formulation performance across development stages is of great importance, but for this purpose, the dissolution method must be able to discriminate between formulations with different quality attributes [30], and show product performance.

Differences in the dissolution profiles of tested drug products may be related to the excipient composition. Therefore, drug performance is greatly influenced by the quality of excipients used in the formulation and manufacturing process [31]. In other cases, these changes in dissolution data may be caused by a lack of robustness rather than product deficiency. However, the selected method must have discriminatory power to detect significant changes between products. It must also be robust to avoid unnecessary product rejection [32].

For fixed-dose combination formulations, this is more complex since for HTZ, as observed in this study, both the United States Pharmacopoeia dissolution method and the proposed method verify that some formulations meet quality specifications as established in the United States Pharmacopoeia [14]. However, the pharmacopeial method is inappropriate for all products evaluated for VAL.

The FDA, EMA and other regulatory agencies recognize several methods to assess the results of dissolution profiles, and multivariate analysis is a tool used when it is necessary to compare objects with similar characteristics [33,34,35].

According to HTZ principal components analysis (PCA) results, three groups were formed (Figure S8). The first two principal components (PC1 and PC2) retain 96.98% of the information contained in original variables (input data), in which it is observed that reference products P2 and C1 form a group with the highest DE values. In addition, B1 and B2 had similar percent of HTZ dissolved within the first 5 min of the assay and formed another group. While for P1, with MDT = 5.82, an individual group was formed, which indicates that this product has a lower dissolution rate.

For VAL, the multivariate analysis also classified products into three groups (Figure S9). Component 1 (PC1) and component 2 (PC2) retained 93.10% of the variance contained in the original input data. The reference drug product was placed in an individual group, with the highest percentage dissolved in 5 min (93%) and the highest dissolution rate with MDT = 2.83 min.

It was observed that there are differences between methods developed for quality control (pharmacopeial method, to assess formulation for batch release) and the method developed to assess product dissolution profile (that provides more accurate information on the formulation performance). However, the latter has proved to be a method that guarantees total HTZ and VAL release from assessed formulations without compromising their discriminative power.

## 4. Conclusions

The developed method proved to be discriminative when assessing dissolution profiles of fixed-dose combination formulations containing HTZ and VAL (12.5 mg + 160 mg) under the following conditions: 900 mL of phosphate buffer pH 6.8 as dissolution medium, 75 rpm of rotation speed, using sinker for 60 min. All fixed-dose combination formulations containing HTZ 12.5 mg + VAL 160 mg sold in drugstores in Brazil and Peru showed similar dissolution profiles for both HTZ and VAL. The approach used in this work allowed us to understand which parameters used in the dissolution test directly influence the release of HTZ and VAL. This strategy can be applied to different drugs, mainly those from BCS classes II and IV, in associations, bringing more assertive tests for comparing different drug products. Additionally, combining statistical experimental design and in silico simulation using DDDPlus™ can minimize the experimental dissolution lab work.

## Figures and Tables

**Table 1:** VAL and HTZ tablet formulations for in vitro and silico dissolution method development.

Formulation Component	F1		F2		F3	
	%	mg	%	mg	%	mg
Valsartan	45.7	160.0	45.7	160.0	45.7	160.0
Hydrochlorothiazide	3.6	12.5	3.6	12.5	3.6	12.5
PH 200 Microcrystalline cellulose	48.7	170.5	--	--	--	--
PH 302 Microcrystalline cellulose	--	--	47.7	167.0	--	--
Croscarmellose Sodium	--	--	1.0	3.5	2.0	7.0
Colloidal silicon dioxide	1.0	3.5	1.0	3.5	1.0	3.5
Lactose monohydrate	--	--	--	--	46.7	163.5
Magnesium Stearate	1.0	3.5	1.0	3.5	1.0	3.5
Total	100.0	350.0	100.0	350.0	100.0	350.0

**Table 2:** Experimental design factors and levels for in vitro dissolution method development.

Factors	Levels		
Rotation speed	50	75	100
Sinker	none	sinker (spring style)	Japanese basket
Formulation	F1	F2	F3

**Table 3:** HTZ and VAL physical-chemical data used for DDDPlus™ simulations.

Physicochemical Information	VAL	HTZ
Dose (mg)	160	12.5
Ingredient type	Active	Active
Molecular weight (g/mol)	435.53 (b)	297.74 (b)
Reference solubility (mg/mL)	0.072 (a)	0.81 (a)
pH for reference solubility	3.77 (b)	5.67 (b)
Density (g/mL)	1.2 (c)	1.2 (c)
Precipitation time	900 (b)	900 (b)
Diffusion coefficient (cm^2^/s × 10^−5^)	0.59 (b)	0.50 (b)
LogP	3.55 (b)	−0.0523 (b)

(a) Experimental data. (b) Calculated from molecules structure by ADMET Predictor^®^ module. (c) Default value.

**Table 4:** HTZ and VAL solubility (LogS and mg/mL) at different pH values via the potentiometric method.

pH	Hydrochlorothiazide		Valsartan	
	LogS	mg/mL	LogS	mg/mL
1.2	−2.587	0.771	−3.908	0.054
3.5	−2.587	0.771	−3.515	0.133
4.5	−2.587	0.771	−2.454	1.530
6.8	−2.580	0.783	1.044	4.821

**Table 5:** Dissolution percentages at 15 min (Q%15), 30 min (Q%30) and dissolution efficiency (DE) for HTZ.

Assay	Formulation	Rotation Speed (rpm)	Sinker	Q%15	Q%30	DE
E1	F1	50	none	16.1 ± 0.2	27.5 ± 0.9	25.2 ± 0.1
E2	F3	50	sinker	94.7 ± 0.5	98 ± 3.84	92.48 ± 0.07
E3	F2	50	Japanese basket	36.0 ± 5	63 ± 7.71	51.59 ± 0.06
E4	F3	75	none	49 ± 0.11	76 ± 4.04	63.68 ± 0.01
E5	F2	75	sinker	40 ± 5.70	79 ± 4.04	60.61 ± 0.03
E6	F1	75	Japanese basket	85 ± 3.16	94 ± 6.94	82.32 ± 0.06
E7	F2	100	none	36 ± 2.76	62 ± 4.57	56.65 ± 0.03
E8	F1	100	sinker	82 ± 1.03	86 ± 0.38	76.84 ± 0.01
E9	F3	100	Japanese basket	88 ± 8.66	89 ± 9.60	83.94 ± 0.01

Results are presented as mean values and standard deviation from three determinations.

**Table 6:** Dissolution percentages at 15 min (Q%15), 30 min (Q%30) and dissolution efficiency (DE) for VAL.

Assay	Formulation	Rotation Speed (rpm)	Sinker	Q%15	Q%30	DE
E1	F1	50	none	12 ± 0.42	21 ± 0.97	19.56 ± 0.01
E2	F3	50	sinker	76 ± 2.57	86 ± 2.42	76.93 ± 0.02
E3	F2	50	Japanese basket	28 ± 4.32	58 ± 4.81	45 ± 0.07
E4	F3	75	none	41 ± 7.74	76 ± 8.10	57.35 ± 0.09
E5	F2	75	sinker	31 ± 6.67	79 ± 5.79	56.79 ± 0.08
E6	F1	75	Japanese basket	71 ± 2.36	81 ± 2.73	70.55 ± 0.01
E7	F2	100	none	34 ± 5.79	60 ± 6.15	58.04 ± 0.04
E8	F1	100	sinker	76 ± 1.09	81 ± 3.10	73.72 ± 0.02
E9	F3	100	Japanese basket	77 ± 1.35	79 ± 0.74	75.14 ± 0.01

Results are presented as mean values and standard deviation from three determinations.

**Table 7:** Dissolution efficiency (DE) and R^2^ values were calculated from simulated dissolution percentages in DDDPlus™ for HTZ and VAL.

Assay Conditions				DE		R^2^	
Run	Formulation	Rotation Speed (rpm)	Sinker	HTZ	VAL	HTZ	VAL
1	F1	50	none	28.6	21.9	0.97	0.96
2	F1	50	sinker	83.6	70.5	0.59	0.60
3	F1	50	Japanese basket	85.2	76.2	0.96	0.93
4	F1	75	none	29.6	22.8	0.97	0.95
5	F1	75	sinker	85.1	72.2	0.62	0.59
6	F1	75	Japanese basket	86.5	76.2	0.95	0.93
7	F1	100	none	28.6	26.6	0.97	0.86
8	F1	100	sinker	87.1	73.7	0.61	0.57
9	F1	100	Japanese basket	87.5	75.4	0.92	0.93
10	F2	50	none	48.5	51.4	0.92	0.98
11	F2	50	sinker	56.7	56.8	0.92	0.96
12	F2	50	Japanese basket	54.2	48.6	0.99	0.98
13	F2	75	none	57.7	54.0	0.99	1.00
14	F2	75	sinker	60.9	57.5	0.96	0.97
15	F2	75	Japanese basket	58.4	51.4	0.97	0.98
16	F2	100	none	55.9	56.1	1.00	0.99
17	F2	100	sinker	64.0	62.3	0.96	0.97
18	F2	100	Japanese basket	61.6	53.6	0.93	0.95
19	F3	50	none	59.9	58.7	0.90	0.92
20	F3	50	sinker	92.7	75.4	0.98	0.61
21	F3	50	Japanese basket	73.5	72.1	0.56	0.59
22	F3	75	none	64.9	61.5	0.94	0.96
23	F3	75	sinker	93.3	85.7	0.94	0.61
24	F3	75	Japanese basket	75.5	74.3	0.53	0.65
25	F3	100	none	68.0	63.6	0.93	0.97
26	F3	100	sinker	93.7	86.5	0.87	0.61
27	F3	100	Japanese basket	79.4	75.2	0.57	0.61

**Table 8:** HTZ and VAL content in formulations marketed in Brazil and Peru.

Product Code	Hydrochlorothiazide			Valsartan		
	mg	%	SD	mg	%	SD
Reference	11.84	94.74	0.66	158.71	99.19	0.39
B1	11.97	95.72	0.14	162.95	101.84	0.09
B2	12.11	96.90	0.26	160.53	100.33	0.35
C1	12.14	97.13	1.35	154.01	97.25	0.34
P1	11.50	92.02	0.37	156.52	108.47	0.51
P2	11.50	91.99	0.41	157.03	98.14	0.79

SD: Standard deviation.

## Data Availability

The data presented in this study are available in the article and the Supplementary Materials.

## Supplementary Materials

The following supporting information can be downloaded at: https://www.mdpi.com/article/10.3390/pharmaceutics15061735/s1, Table S1: Gradient conditions for the mobile phase of the HPLC method; Table S2: Experimental matrix design of the 3^3−1^ fraction factorial design; Table S3: Full 3^3^ factorial design containing the in silico dissolution assays to be used in DDDPlus™ to simulate the dissolution profiles for HTZ and VAL; Table S4: System suitability for HTZ and VAL; Table S5: Descriptive measure of fit quality; Table S6: Cochran test to assess homoscedasticity; Table S7: Results of the precision analysis; Table S8: Accuracy results; Figure S1: Particle size distribution for HTZ (A) and VAL (B); Figure S2: Observed and predicted residues from in vitro dissolution efficiency (DE) for HTZ (A) and VAL (B); Figure S3: Observed and predicted residues from in silico dissolution efficiency (DE) for HTZ (A) and VAL (B); Figure S4: Pareto chart of the linear and quadratic effects of the factors (1) formulation, (2) rotation speed and (3) sinker on in silico dissolution efficiency (DE) for HTZ (A) and VAL (B); Figure S5: Means plot of in silico dissolution efficiency (DE) calculated for HTZ (A) and VAL (B); Figure S6: Surface response plots containing the evaluation of dissolution efficiency (DE) in function of the factors rotation speed and formulation for HTZ (A) and VAL (B), and DE value in function of the factors sinker and rotation speed for HTZ (C) and VAL (D); Figure S7: Formulations F1, F2, and F3 grouped according the Tukey test performed for the DE means for HTZ (A) and VAL (B); Figure S8: Principal component analysis performed using dissolution efficiency (DE) data, mean dissolution time (MDT) and percent of HTZ dissolved between 5 and 60 min of dissolution test for HTZ. Distribution of original variables (A) and products (B); Figure S9: Principal component analysis performed using dissolution efficiency (DE) data, mean dissolution time (MDT), and percent of VAL dissolved between 5 and 60 min of a dissolution test for VAL. Distribution of original variables (A) and products (B); Figure S10: Chromatogram obtained from the analysis of VAL and HTZ; Figure S11: Linearity diagram for HTZ (A) and VAL (B) with adjusted residual values.
