# Supplementary material for: Development of a Discriminative Dissolution Method, Using In-Silico Tool for Hydrochlorothiazide and Valsartan Tablets
Source: Pharmaceutics. 2023 Jun 14;15(6):1735. doi: 10.3390/pharmaceutics15061735 (PMC10301187; doi:10.3390/pharmaceutics15061735)
Supplement: Supplementary file 1 [file pharmaceutics-15-01735-s001.zip › pharmaceutics-2374664-supplementary.pdf]

Table S1. Gradient conditions for the mobile phase of the HPLC method

Time (min)	Mobile phase flow (mL/min)	0.01 M ammonium formate (%)	Acetonitrile (%)
0	0.9	15	85
3.7	0.9	95	5
4.5	0.9	95	5
4.6	0.9	15	85
12.0	0.9	15	85

Table S2. Experimental matrix design of the 3^{3-1} fraction factorial design

Run	Rotation speed (rpm)	Sinker	Formulation
1	50	None	F1
2	50	Sinker	F3
3	50	Japanese basket	F2
4	75	None	F3
5	75	Sinker	F2
6	75	Japanese basket	F1
7	100	None	F2
8	100	Sinker	F1
9	100	Japanese basket	F3

Table S3. Full 3^3 factorial design containing the in silico dissolution assays to be used in DDDPlus™ to simulate the dissolution profiles for HTZ and VAL

	Assay conditions		
**Run**	**Formulation**	**Rotation speed (rpm)**	**Sinker**
1	F1	50	none
2	F1	50	sinker
3	F1	50	Japanese basket
4	F1	75	none
5	F1	75	sinker
6	F1	75	Japanese basket
7	F1	100	none
8	F1	100	sinker
9	F1	100	Japanese basket
10	F2	50	none
11	F2	50	sinker
12	F2	50	Japanese basket
13	F2	75	none
14	F2	75	sinker
15	F2	75	Japanese basket
16	F2	100	none
17	F2	100	sinker
18	F2	100	Japanese basket
19	F3	50	none
20	F3	50	sinker
21	F3	50	Japanese basket
22	F3	75	none
23	F3	75	sinker
24	F3	75	Japanese basket
25	F3	100	none
26	F3	100	sinker
27	F3	100	Japanese basket

Figure S1. Particle size distribution for HTZ (A) and VAL (B)

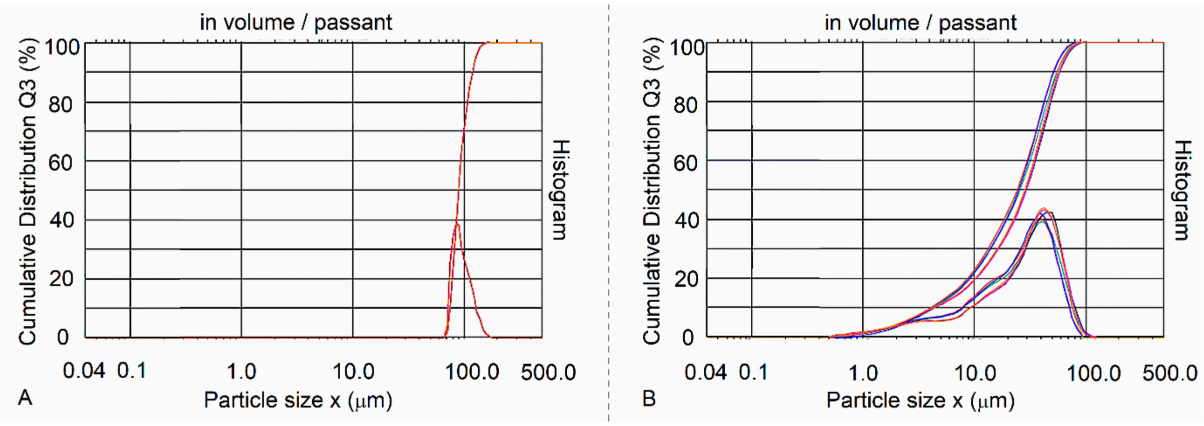

Figure S2. Observed and predicted residues from in vitro dissolution efficiency (DE) for HTZ (A) and VAL (B)

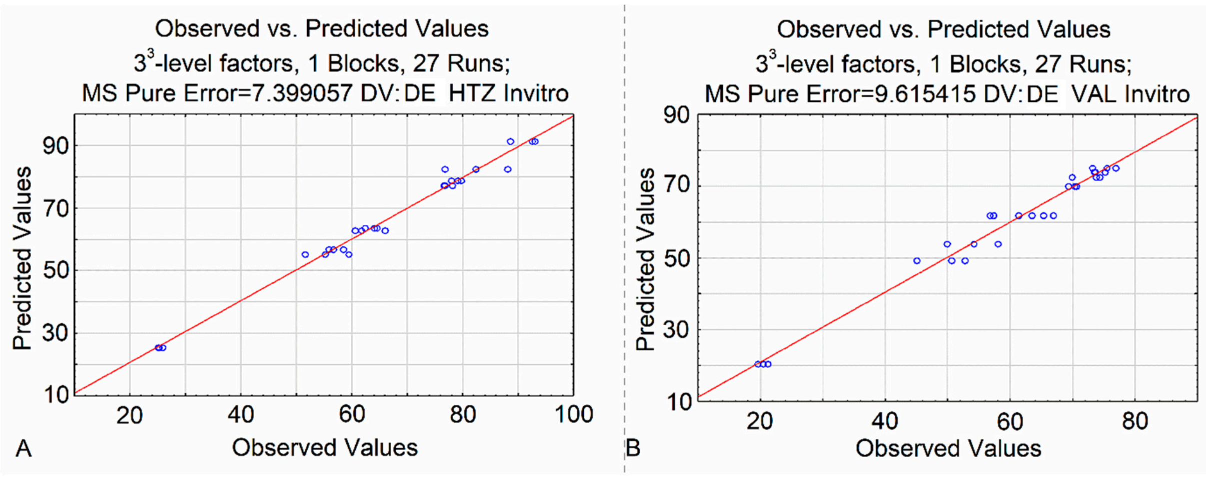

Figure S3. Observed and predicted residues from in silico dissolution efficiency (DE) for HTZ (A) and VAL (B)

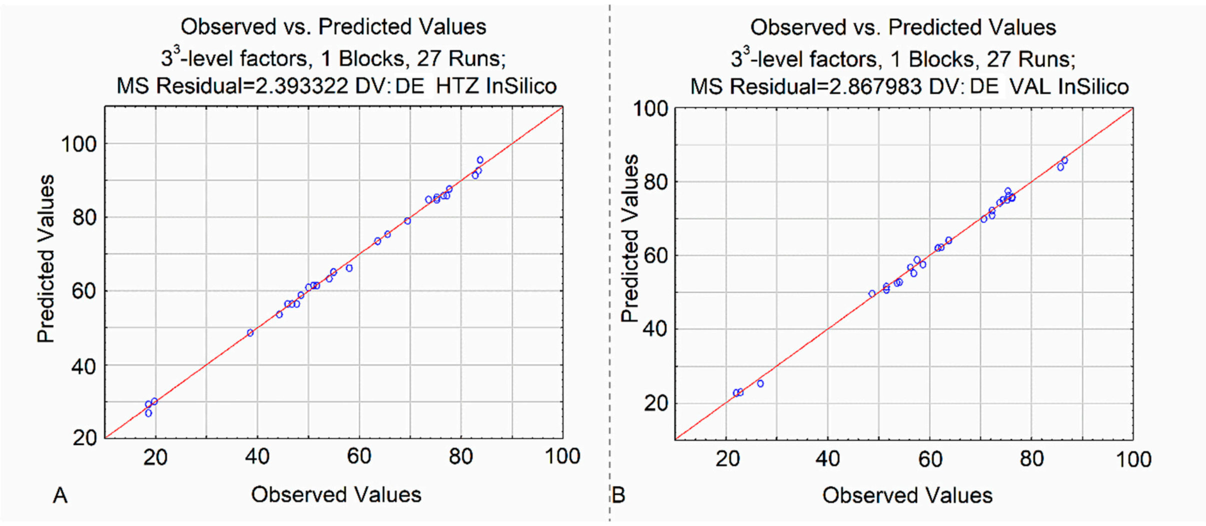

Figure S4. Pareto chart of the linear and quadratic effects of the factors (1) formulation, (2) rotation speed and (3) sinker on in silico dissolution efficiency (DE) for HTZ (A) and VAL (B)

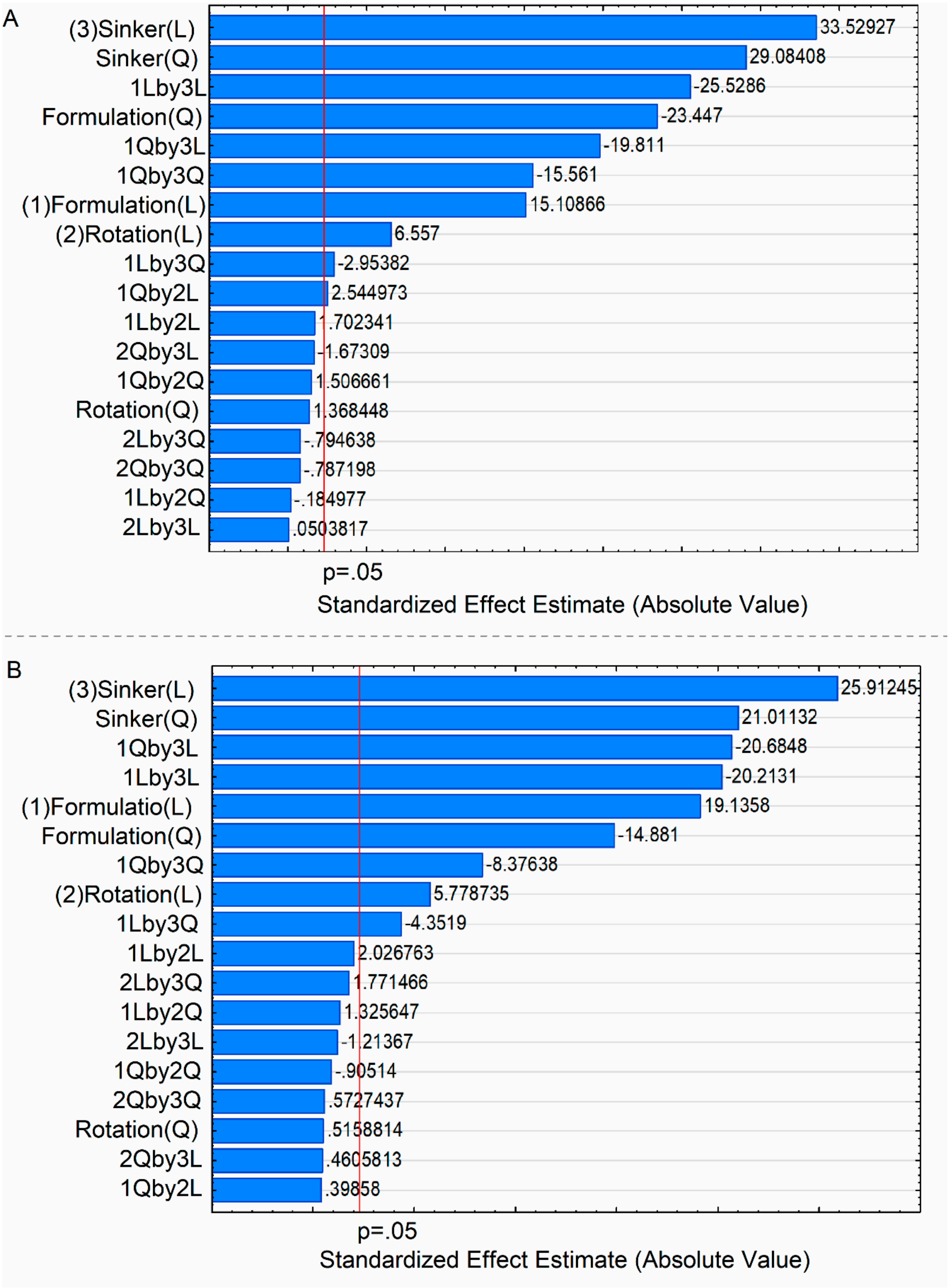

Figure S5. Means plot of in silico dissolution efficiency (DE) calculated for HTZ (A) and VAL (B)

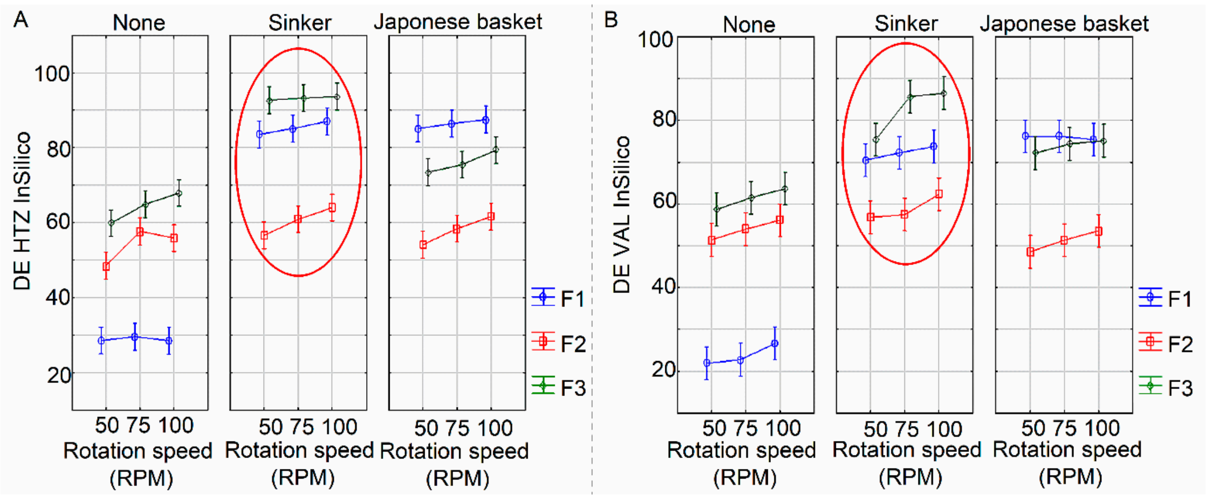

Figure S6. Surface response plots containing the evaluation of dissolution efficiency (DE) in function of the factors rotation speed and formulation for HTZ (A) and VAL (B), and DE value in function of the factors sinker and rotation speed for HTZ (C) and VAL (D)

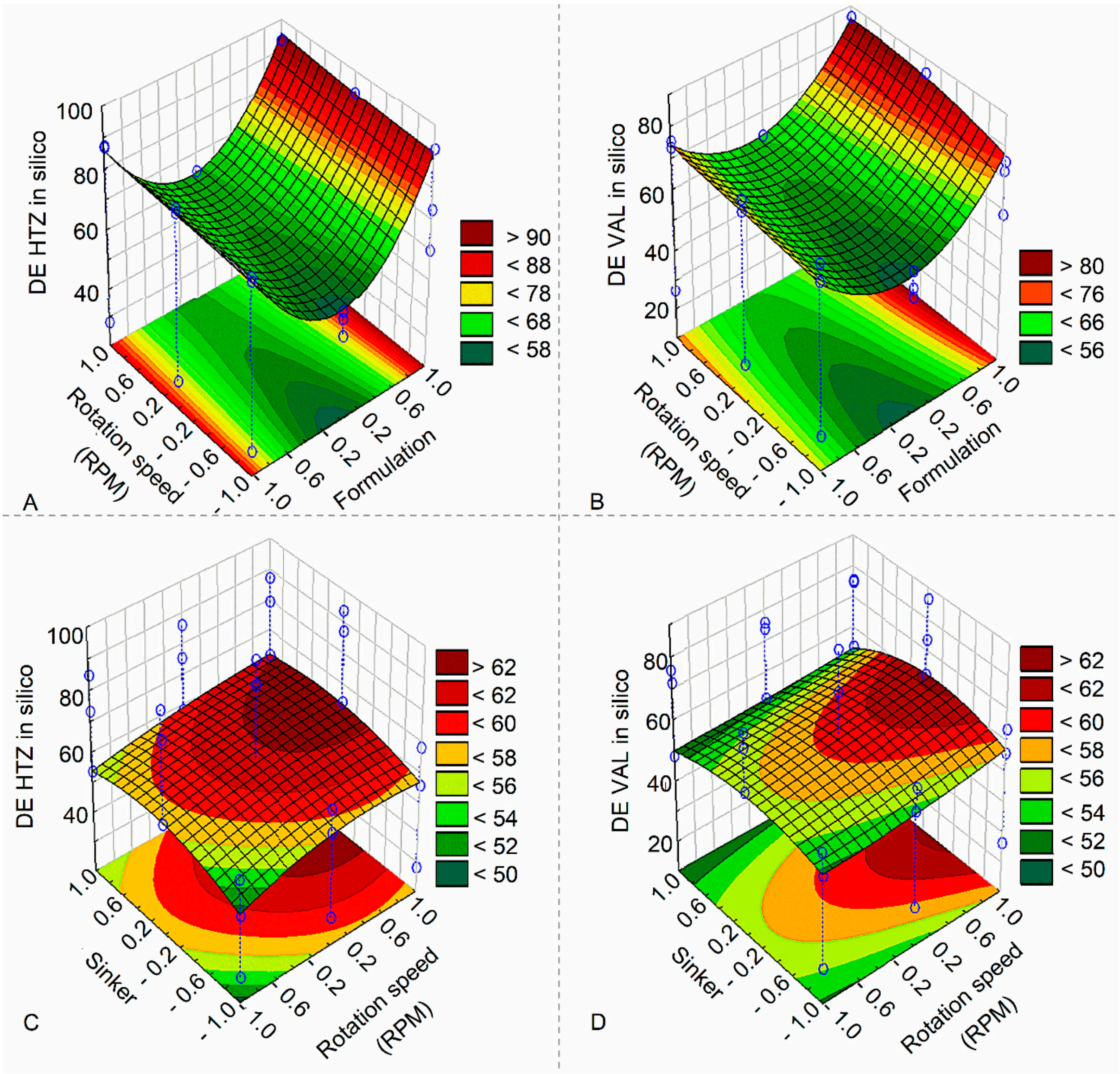

Figure S7. Formulations F1, F2, and F3 grouped according to the Tukey test performed for the DE means for HTZ (A) and VAL (B)

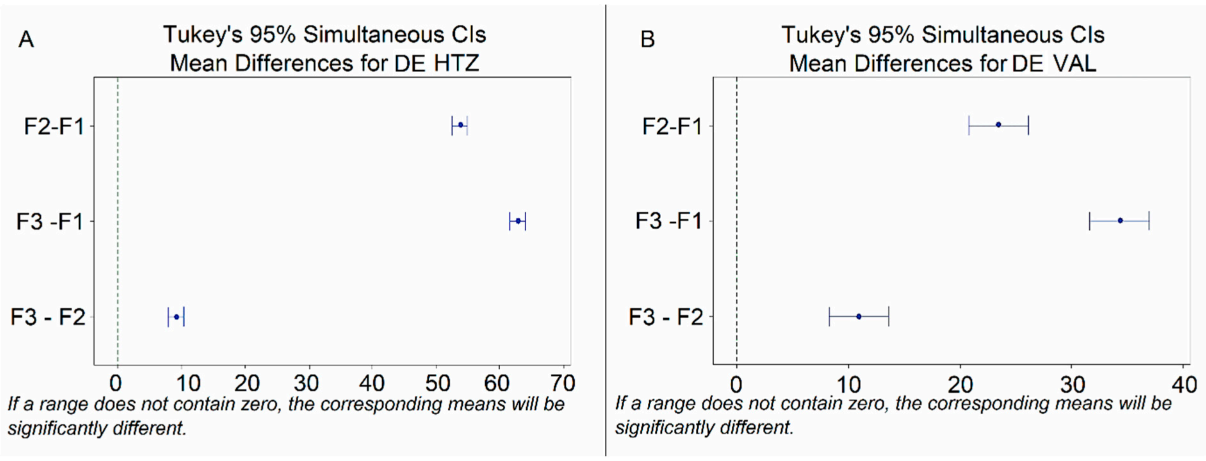

Figure S8. Principal component analysis performed using dissolution efficiency (DE) data, mean dissolution time (MDT) and percent of HTZ dissolved between 5 and 60 minutes of dissolution test for HTZ. Distribution of original variables (A) and products (B).

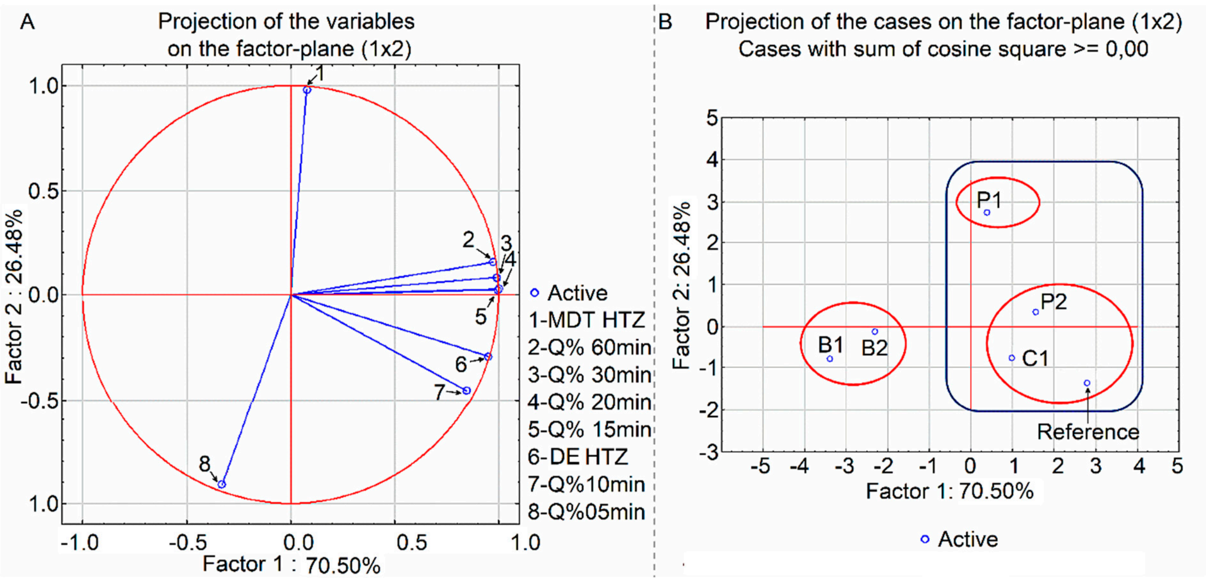

Figure S9. Principal component analysis performed using dissolution efficiency (DE) data, mean dissolution time (MDT) and percent of VAL dissolved between 5 and 60 minutes of dissolution test for VAL. Distribution of original variables (A) and products (B).

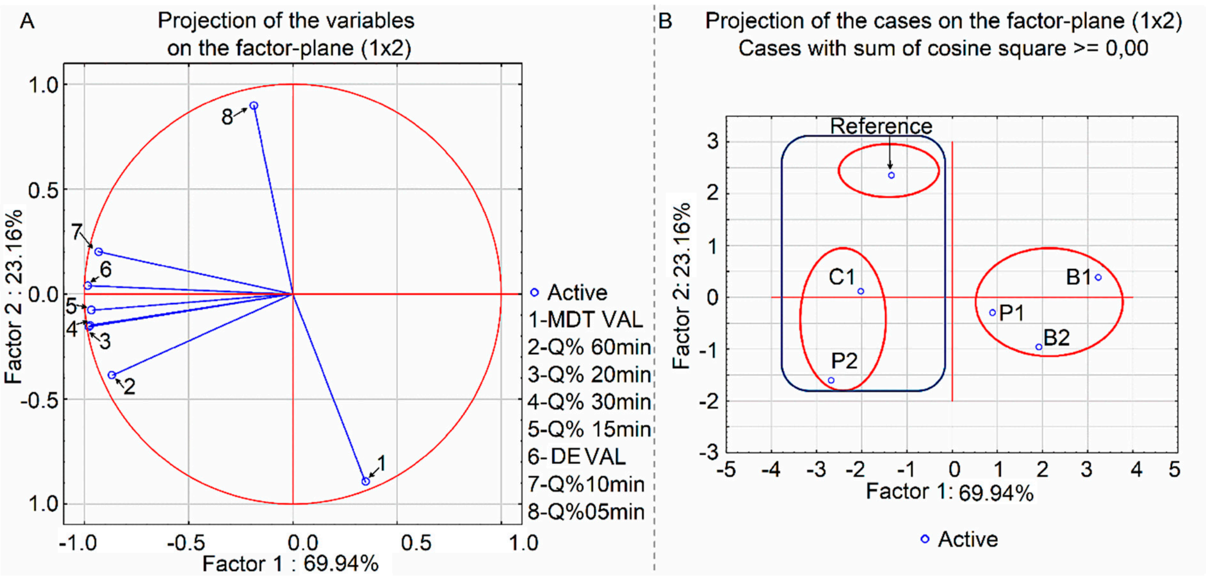

UHPLC Validation Data

The software Fusion Method Development™ (S-Matrix, California, USA) was used for the UHPLC method development and to process the results. The software Action Stat (Estatcamp, São Carlos, Brazil) was used for statistical analysis. The validation parameters are briefly described below.

Selectivity

It was evaluated by comparing the chromatograms of the blank solution (mobile phase) and buffer solutions used as dissolution media, and matrix stock solution (formulation containing VAL and HTZ). The chromatogram showing the selectivity of the method is shown in Figure S10.

It was observed that there was no interference in the chromatogram (Figure S10) for VAL and HTZ in the dissolution samples at different pH conditions.

Figure S10. Chromatogram obtained from the analysis of selectivity of VAL and HTZ.

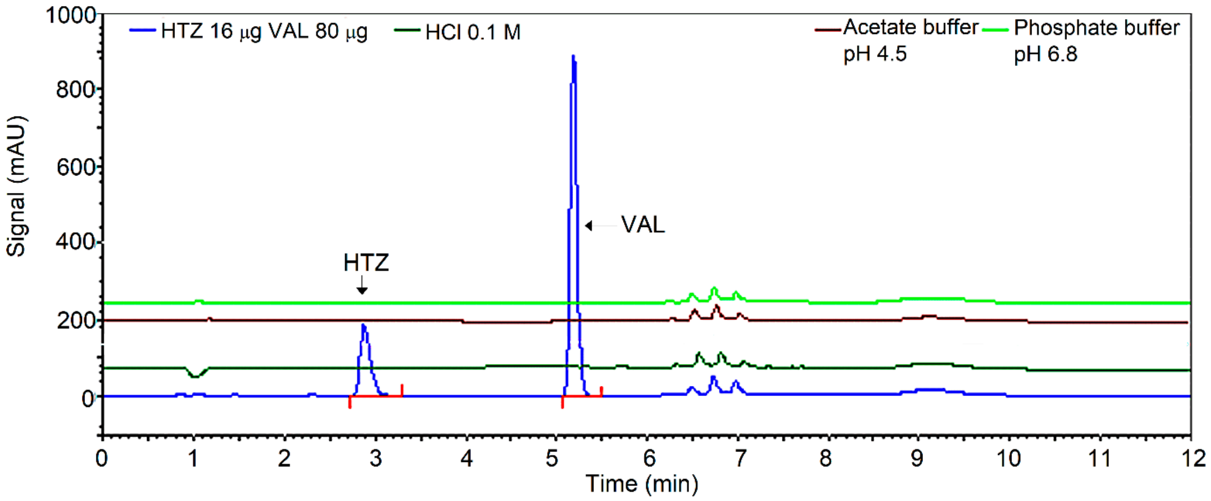

System suitability

It was evaluated in the validation stage of the method, by analyzing six replicates of a standard solution. The results are presented in Table S4.

Table S4. System suitability for HTZ and VAL.

Sample	Asymmetry	Theoretical plates	Capacity factor	Resolution
HTZ	1.42	3714	3.42	--
VAL	1.36	24188	6.24	12

Linearity

The calibration curves (Figure S11) were obtained in the range of drug concentrations: 2 – 28 μg/mL for HTZ and 10 – 170 μg/mL for VAL, The R^2 values were 0.9969 for HTZ and 0.9992 for VAL, indicating that there is adequate linear correlation between both drug concentrations and the chromatographic areas. The residual evaluation showed normal distribution of the data and homoscedastic for both drugs (Tables S5 and S6).

Figure S11. Linearity diagram for HTZ (A) and VAL (B) with adjusted residual values

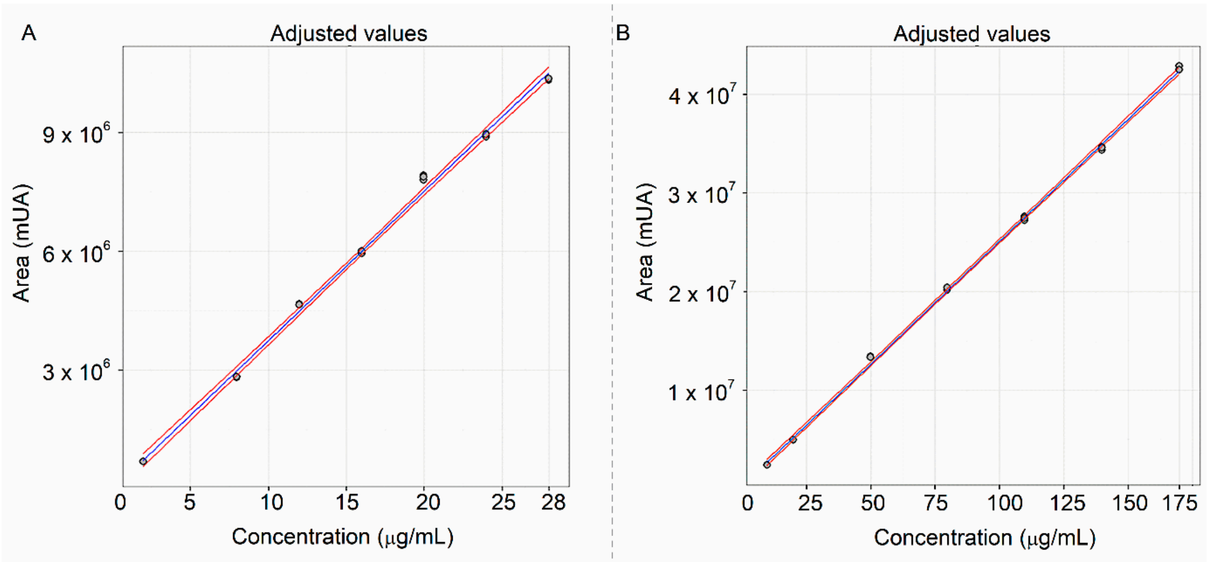

Table S5. Descriptive measure of fit quality.

Sample	Standard deviation of residuals	Degrees of freedom	R^2	Coefficient of correlation
HTZ	187372.61	19	0.9969	0.9984
VAL	413728.33	19	0.9992	0.9996

Table S6. Cochran test to assess homoscedasticity.

Sample	Statistics	Degrees of freedom	p-value
HTZ	0.3847	7	1
VAL	0.4563	7	0.6284

Precision

It was assessed in 9 determinations covering the specified range detailed in linearity test, using 3 concentrations / 3 replicates, considering an acceptable variation for intermediate precision.

The data obtained in the precision analysis are shown in Table S7. The values of the relative standard variation (RSD) for both intraday and intermediate precision were less than 2.0%, which demonstrates that the method is sufficiently accurate.

Table S7. Results of the precision analysis.

Sample	Concentration (µg/mL)	Intraday precision		Intermediate precision	
		Mean area (mAU) ± SD	RSD	Mean area (mAU) ± SD	RSD
HTZ	2.0	474899 ± 3485	0.73	462815 ± 1065	0.23
	16.0	4924978 ± 19327	0.39	4860806 ± 42225	0.87
	28.0	7619371 ± 20472	0.27	8004142 ± 25591	0.32
VAL	10.0	2619382 ± 39792	1.52	2529530 ± 1033	0.04
	80.0	21861795 ± 43127	0.19	21535170 ± 261471	1.21
	170.0	42896379 ± 354797	0.83	42506256 ± 122601	0.29

Accuracy

Accuracy evaluates the recovery percentages from a sample enriched with standard solution. It was assessed in 9 determinations in 3 concentration levels covering the specified range. An average recovery of 97.84% for HTZ and 102.89% for VAL was obtained as shown in Table S8, meeting the accuracy acceptance criteria.

Table S8. Accuracy results

Parameters	HTZ	VAL
Recovery (%)	97.84	102.89
Standard deviation	3.08	2.19
Degrees of freedom	8	8
Lower limit (%)	95.93	101.53
Upper limit (%)	99.75	104.25
Specified lower limit (%)	95	95
Specified upper limit (%)	105	105
